# Cerebral dopamine neurotrophic factor (CDNF) protects against quinolinic acid-induced toxicity in in vitro and in vivo models of Huntington’s disease

**DOI:** 10.1038/s41598-020-75439-1

**Published:** 2020-11-05

**Authors:** P. Stepanova, V. Srinivasan, D. Lindholm, M. H. Voutilainen

**Affiliations:** 1 Institute of Biotechnology, HiLIFE, University of Helsinki, BO Box 56, 00014 Helsinki, Finland; 2 Medicum, Department of Biochemistry and Developmental Biology, Faculty of Medicine, University of Helsinki, PO Box 63, 00014 Helsinki, Finland; 3 Minerva Foundation Institute for Medical Research, Biomedicum Helsinki 2U, Tukholmankatu 8, Helsinki, Finland

**Keywords:** Huntington's disease, Neurotrophic factors

## Abstract

Huntington’s disease (HD) is a neurodegenerative disorder with a progressive loss of medium spiny neurons in the striatum and aggregation of mutant huntingtin in the striatal and cortical neurons. Currently, there are no rational therapies for the treatment of the disease. Cerebral dopamine neurotrophic factor (CDNF) is an endoplasmic reticulum (ER) located protein with neurotrophic factor (NTF) properties, protecting and restoring the function of dopaminergic neurons in animal models of PD more effectively than other NTFs. CDNF is currently in phase I–II clinical trials on PD patients. Here we have studied whether CDNF has beneficial effects on striatal neurons in in vitro and in vivo models of HD. CDNF was able to protect striatal neurons from quinolinic acid (QA)-induced cell death in vitro via increasing the IRE1α/XBP1 signalling pathway in the ER. A single intrastriatal CDNF injection protected against the deleterious effects of QA in a rat model of HD. CDNF improved motor coordination and decreased ataxia in QA-toxin treated rats, and stimulated the neurogenesis by increasing doublecortin (DCX)-positive and NeuN-positive cells in the striatum. These results show that CDNF positively affects striatal neuron viability reduced by QA and signifies CDNF as a promising drug candidate for the treatment of HD.

## Introduction

Huntington’s disease (HD) is a hereditary, progressive neurodegenerative disorder that exhibits motor and non-motor symptoms. HD is associated with an expansion of CAG trinucleotide repeats in exon 1 of the *huntingtin* gene, which encodes for an elongated polyglutamine (PolyQ) stretch in Huntingtin protein (Htt)^1^, resulting in expression of mutant Htt with varying degree of CAG repeats (more than 36 to 120 repeats). Htt is expressed at high levels in striatal neurons^2^, which are the most vulnerable to degeneration in HD. Mutant Htt is prone to misfolding and aggregation into soluble oligomers, which involve cytotoxicity and leads to neurodegeneration in HD^3^. Moreover, soluble inclusions are forming insoluble huntingtin inclusions^4^. These insoluble aggregates result in enhanced cell degeneration via altered gene transcription, impaired cell signalling, increased endoplasmic reticulum (ER) stress and mitochondrial dysfunctions^5–7^. The selective and progressive degeneration of cells in the caudate and putamen (striatum), particularly the medium spiny neurons (MSNs), is a characteristic for HD. This is accompanied by increase in neuroinflammation and loss of trophic support like, reduced expression of the neurotrophin and brain-derived neurotrophic factor (BDNF) in the dorsal striatum in HD patients^8^. Previous studies showed that wild-type huntingtin but not mutant increased BDNF production and is involved in BDNF vesicle transport in vitro and in vivo^9–11^. Furthermore, the extension of caudate atrophy in HD leads to a cognitive impairment such as mental processing and mood^12^. The majority of drugs, which were successful in pre-clinical trials of HD, have failed to delay the progression of disease in HD patients^13^. Currently, “huntingtin lowering” strategies using antisense oligonucleotide (ASO), have been developed for clinical trials of HD. Among them, the most perspective one is based on the intrathecal delivery and using whole Htt as the main target^14^. Htt is involved in the normal neuronal development^15^, and the effect of partial suppression of it in long-term perspective is still unclear. A dual strategy using ASO in combination with drugs or other factors may prove beneficial for the treatment of HD.

Neurotrophic factors are secretory proteins that promote the survival and differentiation of neurons both during development and in different disease conditions and influence different intracellular signalling pathways that counteract the process of neuronal degeneration^16^. Different neurotrophic factors, such as BDNF, ciliary neurotrophic factor (CNTF), insulin-like growth factor-I (IGF-I), nerve growth factor (NGF), neurturin (NRTN) and glial cell line-derived neurotrophic factor (GDNF) have demonstrated a protective role in genetic and neurotoxin models of HD including the 2,3‐pyridine dicarboxylic acid (quinolinic acid, QA) and 3-nitropropionic acid (3-NP) models^17–23^.

Of the neurotoxins applied as HD models, QA is an NMDA agonist that induces excitotoxic lesions in the striatum, mainly in MSNs^24^. In addition to neurons, activated macrophages and microglial cells produce QA at neurotoxic concentrations in the brain^25–27^. QA induces neurotoxicity by multiple mechanisms following the over-activation of NMDA-receptors, including enhanced oxidative stress, changes in cell signalling and secondary mitochondrial deficits. Injections of QA into the striatum of rodents or primates has shown to produce similar neurochemical features as seen in HD patients^28,29^, making it a useful tool to study the neuropathology of HD^30^.

CDNF is an endoplasmic reticulum (ER) located protein with neurotrophic factor (NTF) properties present in different tissues including striatum and cortex in the brain^31–34^. CDNF consists of two structural domains, a saposin-like N-terminal lipid-binding domain, and the carboxy-terminal SAP-like domain, containing a conserved CXXC motif and ending with ER retentions signal^35,36^. CDNF differs from the classical NTFs, having a novel mechanism of action and structure. CDNF is thought to act by regulating the unfolded protein response (UPR) intracellularly but is also secreted from the cells during ER stress^37^. Furthermore, CDNF inhibits the synthesis and release of pro-inflammatory cytokines^38^.

Previous studies from our laboratory have shown that CDNF promotes recovery and survival of midbrain dopamine neurons in vivo in rodent and primate models of Parkinson’s diseases (PD)^31,39,40^. Currently, CDNF is in a first-in-human Phase 1–2 clinical trials as a promising factor to counteract midbrain cell degeneration and the progression of PD in humans. The first results revealed the safety and no side effects of CDNF in PD patients at advanced-stages^41^. (EudraCT 2015-004175-73; ClinicalTrials.gov: NCT03295786).

The pathways and mechanisms for cell demise may be partly similar in different neurodegenerative disorders warranting studies of CDNF in HD. In this work, we evaluated the role of CDNF against the deleterious effects of QA in vitro and in vivo after a single unilateral injection of CDNF into the striatum. We observed that CDNF effectively protected striatal neurons against cell degeneration induced by QA via affecting the IRE1α signalling pathway shown to be protective in models of HD^42^. This underscores the potential of CDNF as a promising factor in the future treatment of HD.

## Material and methods

### Subjects

Adult male Wistar rats (250–300 g) were used in the CDNF diffusion experiment and QA experiment. The rats were randomly housed in groups of four-five animals per cage in a temperature and humidity-controlled facility maintained on a 12:12 light/dark cycle with free access to food and water throughout the experiment. The design of animal experiments was approved by the National Animal Experiment Board of Finland. The laboratory animal experiment protocol approval number was ESAVI/7812/04.10.07/2015.

### Administration of CDNF in diffusion experiments

**Figure 1:**
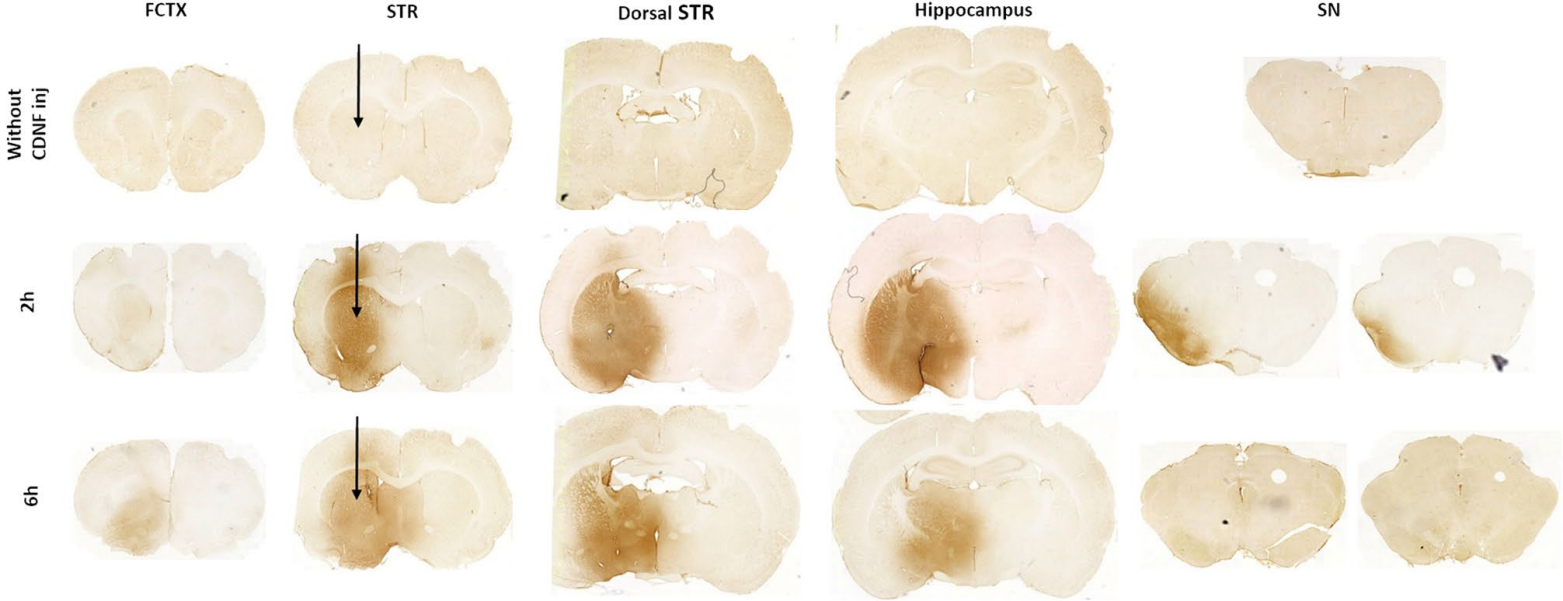
Diffusion of CDNF in rat brain tissue. Rats were injected with 10 μg CDNF or PBS injection into the same injection site as used in QA model. Immunohistochemical staining of CDNF illustrating the distribution of the neurotrophic protein in frontal cortex (FCTX), striatum (STR), dorsal striatum, hippocampus and substantia nigra (SN) in 2 or 6 h after single intrastriatal injection. The black arrow indicates the placement of intrastriatal injection. Images were taken with Pannoramic 250 digital slide scanner (3DHISTECH, Hungary).

In the diffusion experiment (Fig. 1), naïve rats received a single unilateral intrastriatal injection of CDNF (10 μg in 4 μl) (human recombinant CDNF, Batch 00400, 9.6 µg/µl, Biovian, Turku, Finland).The injection speed was 0.5 μl/min with Hamilton syringe (0.46 mm). The needle was left in place for 4 min after each injection to allow diffusion and to minimize the backflow of the solution. Rats were perfused either 2 or 6 h after injection.

### Stereotaxic injection of QA and CDNF in neuroprotection experiment

**Figure 2:**
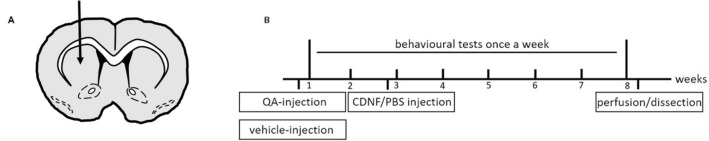
Study design. Intrastriatal vehicle (PBS) injection or 225 nM QA injection, which was followed by 10 μg CDNF or PBS into the same injection site in 2 weeks post-lesion. Behavioural tests were conducted weekly until the end of the study. Animals were sacrificed for histological analyses after 8 weeks post-lesion. The schemes were created with PowerPoint Microsoft Office 2016 program.

All stereotaxic and surgical procedures were performed under isoflurane anaesthesia (4.5% during induction and 3% during maintenance). Rats received one unilateral intrastriatal microinjection of QA (Tocris, UK, 2 μl, 225 nM concentration) (A/P + 0.7, M/L + 2.8, D/V − 6.0), according to the rat brain atlas^43^ (Fig. 2A). The optimal concentration of QA was chosen based on the results from previous studies^44–46^. QA was injected at the rate of 0.5 μl/min with Hamilton syringe (0.46 mm). After injection, the needle was kept in place for 5 min to prevent the backflow of the solution. Animals from the vehicle group received one intrastriatal injection of phosphate-buffered saline (PBS). QA-lesioned rats received a single unilateral intrastriatal injection of CDNF (10 μg/4 μl) (human recombinant CDNF, Batch 00400, 9.6 µg/µl, Biovian, Turku, Finland) or phosphate-buffered saline (PBS) 2 weeks post-lesion. The concentration for CDNF was selected based on the previous in vivo studies in 6-OHDA rat model of PD^31^. Rats were balanced based on their motor behaviour in rotarod tests before CDNF and PBS injection. The injection speed was 0.5 μl/min with Hamilton syringe (0.46 mm). The needle was left in place for 4 min after each injection to allow diffusion and to minimize the backflow of the solution. Behavioural tests were conducted weekly. All rats were sacrificed after 8 weeks post-lesion, and their brains were perfused for histological analyses or dissected for western blot analysis (Fig. 2B).

### Behavioural studies

Rats were habituated for the behavioural tests before the actual experiment. The locomotor activity of the rats was measured using the Rotarod test system (Ugo Basile, Italy). More specifically, motor behaviour was analyzed once a week based on the duration of the balancing on the Rotarod platform with speed 4–40 rpm (revolutions per minute) during a maximum of 120 s. Additionally, the grip strength test (Chatillon Force Measurement Products, USA) was used to measure neuromuscular function and the strength of forelimbs. The strength of left and right forelimbs were measured separately. Animals were allowed to grip the metallic grid. The maximum peak force from three trials was recorded. Furthermore, the balance beam test was applied as an additional test to measure motor coordination. Animals were placed on the round wooden stick (3 cm in diameter) with the dark goal box at the end of the stick. The task of the animals was to reach the goal box with 3 min time limit. The number of paw slips and latency to walk was recorded. Digigait test (Ventral Plane Imaging (VPI) Technology, USA) was used to perform a gait analysis during animals’ treadmill walking. To measure gait analysis, rats were placed individually in a plastic cage with a treadmill belt on the floor. The speed of the treadmill belt during the testing was at a speed of 19 cm/s with zero degrees of elevating, which were the optimal parameters that all animals could perform the test. Approximately 3 s of video from each animal were used in the analysis. During Digigait test, there were missed measurements because of severe condition of animals (six animals in the PBS-treated group with 12 measurements in total, one animal in CDNF-treated group with two missed measurements in total). We estimated missing data as an average of before and after measurements.

### Immunostaining

#### CDNF-staining

For diffusion experiments (Fig. 1), immunostaining was done on free-floating brain sections using the ABC method. Free-floating sections were rinsed with TBS and heated in 10 mM citrate buffer, pH 6, with 0.05% Tween 20 at + 80 °C for 30 min, after they were blocked with 2% normal goat serum (S1000, Vector) in TBS-T (0.015% serum in TBS-T) for 20 min. Sections were then incubated in rabbit anti-human CDNF antibody (stock solution 0.4 mg/ml, Icosagen, 1:500) overnight at + 4 °C. Sections were rinsed in TBS-T (TBS, 0.1% Tween 20) and incubated in biotinylated goat anti-rabbit IgG (1:200, Vector Laboratories, Burlingame, CA) for 1 h in the room temperature, followed by incubation for 30 min with avidin–biotin-horseradish peroxidase complex (ABC kit; Vector) and lastly reacted with 40 s–1 min with 3,3′-diaminobenzidine tetrahydrochloride (DAB) (SK-4100, Vector). Sections were then mounted on slides with coverslips.

#### NeuN- and DCX-staining

After the behavioural experiments, rats were deeply anaesthetised with pentobarbital and perfused with PBS, followed by 4% paraformaldehyde (PFA) in PBS and stored in 20% sucrose. The brains were cut into 40 μm thick sections in a series of six. Immunostaining was done on free-floating brain sections using the ABC method. Free-floating sections were rinsed with PBS. Sections for NeuN-staining were heated in 10 mM citrate buffer, pH 6, for 10 min at + 95 °C after they were blocked with 2% normal goat serum and 0.1% Triton-X. Sections for DCX-staining were suppressed endogenous peroxidase activity in 0.3% hydrogen peroxide (Sigma Aldrich). Then sections were incubated for 1 h with blocking solution (4% BSA, 0.3% Triton-x-100 in PBS). Then sections were incubated in mouse anti-NeuN antibody (Chemicon, #MAB377, 1:200) or goat anti-DCX antibody (1:500, Santa Cruz Biotechnology, #A0614) overnight at + 4 °C. The primary antibody was visualised using biotinylated goat anti-mouse IgG (1:250, Vector Laboratories, Burlingame, CA) for 1 h in the room temperature, followed by incubation for 30 min with avidin–biotin-horseradish peroxidase complex (ABC kit; Vector). Staining was developed 40 s–1 min with DAB (0.5 mg/ml). Sections were mounted on slides with coverslips. Brain sections were analysed from images taken with Pannoramic 250 digital slide scanner (3DHISTECH, Hungary). NeuN-positive cell density from striatum was measured from four to six brain sections and quantitated relative to the control group. For the quantitation of NeuN-positive cells, the Image Pro program was applied, and all measurements were done by personnel blinded as to treatment. The number of NeuN positive cells was determined as a number of clusters of pixels of the default area. The data is presented as a percentage of the non-lesion side. DCX-positive cell from SVZ was measured from four to six brain sections and quantitated relative to the control non-lesion side by the Matlab method^47^. The algorithm recognised the cells based on their intensity and size. The threshold for the size of a single cell was also set manually. The number of DCX positive cells was obtained as a percentage of the non-lesion side.

### Immunoblotting

The rat striatum was dissected on dry ice. Samples were homogenised and lysed in HEPES buffer (pH 7.4) containing protease (cOmplete tablets, EDTA-free EASYpack, Roche) and phosphatase (PhosSTOP EASYpack, Roche) inhibitors. Lysates were then centrifuged at 12.28123×*g* (Heraeus Pico17 Centrifuge, Thermo Scientific), and protein-containing supernatants were collected. Protein samples were diluted in Laemmli’s loading buffer and heated at + 95 °C for 10 min. Twenty micrograms of protein were electrophoresed in polyacrylamide gels (4–15% Mini-PROTEAN TGX precast protein gels, Bio-Rad) and blotted on Nitrocellulose Blotting membrane (Amersham Protran, 0.45 μm). Membranes were blocked in 5% skimmed milk buffered in TBS-T (TBS, 0.1%Tween 20) or 5% BSA in TBS-T and incubated overnight with primary antibodies against DARPP-32 (1:50,000, Abcam, #ab40801) and Tubulin (1:30,000, Sigma, #T9026). After several washes in TBS-T, the blots were incubated for 1 h in horseradish peroxidase-conjugated secondary antibodies (1:3000, Polyclonal goat anti-mouse IgG, Dako, Denmark; donkey anti-rabbit IgG, GE Healthcare, UK). Membranes were rewashed several times and treated with peroxidase substrate (Pierce Western Blotting Substrate, Thermo Scientific, USA). The blots were developed in the imaging system (Optimax Film Processor Machine). The density of blots was measured by ImageJ software and expressed as a ratio of injected and intact side, which were normalized to the loading control.

### Cell cultures

Striatal cells derived from mice and immortalized with a temperature-sensitive large T antigen were used for this study^48^. Cells were grown at 33 °C in Dulbecco’s Modified Eagle’s medium (DMEM, Invitrogen) supplemented with 10% fetal bovine serum. For the experiments, the cells were incubated at 37 °C to induce neuronal differentiation. Cell viability was assayed essentially as described previously^49^ using the MTT method that assesses mitochondrial linked reductive capacity of the cells. In brief, striatal cells, expressing 7Q repeats containing Htt protein, were treated with 3 mM QA for 24 h, and cell death was measured by utilizing tetrazolium dye 3-(4,5-dimethylthiazol-2-yl)-2,5-diphenyltetrazolium bromide (MTT, Sigma) substrate added to cells for two hours. The insoluble formazan substrate was reconstituted in a solvent of 0.1 M HCl-isopropanol and incubated for 30 min with gentle agitation. Absorbance was measured at 560 nm, and the value reflected the relative number of surviving cells following each treatment.

### Immunoblotting from striatal neuronal cell culture

Following the treatments with QA and CDNF, striatal neuronal cultures were harvested for analysis by immunoblotting as previously described^7,50^. Briefly, the cells were washed twice with ice-cold PBS and lysed in RIPA buffer (150 mM NaCl, 1% Triton-X-100, 0.5% sodium deoxycholate, 1% SDS, 50 mM Tris–HCl, pH 7.4) supplemented with protease inhibitor (Roche) and phosphatase inhibitor (Phosphostop, Roche). The lysates were then centrifuged at 16,200×*g* (Heraeus Fresco21 centrifuge, Thermo Scientific) for 15 min, 4 °C and supernatants were collected. Protein concentration measurements were performed with BCA protein assay kit (Pierce, Thermo Fisher Scientific), and equal amounts of protein were loaded onto SDS-PAGE and blotted onto nitrocellulose membrane filters. Following this, the membranes were washed with TBS-T (50 mM Tris–HCl pH 7.5, 150 mM NaCl, 0.1% Tween 20) and blocked for 1 h in 5% skimmed milk or 5% bovine serum albumin, in TBS-T. The membranes were then incubated with the following primary antibodies overnight at 4 °C with gentle agitation, anti-XBP1 (1:1000, Cell signaling technologies, D2C1F); Anti-IRE1α (phosphor S724; 1:2500, Abcam, ab48187), Anti-IRE1α (1:2500, Abcam, ab37073), Anti-GAPDH (1:5000, Millipore, MAB374). Following washes with TBS-T, the membranes were incubated with horseradish peroxidase-conjugated secondary antibodies (1:2500, Jackson Immunoresearch Laboratories) at room temperature for 1 h with gentle agitation. Proteins were detected using enhanced chemiluminescence substrate (Pierce, Thermo Fisher Scientific) and imaged on FLA-9000 scanner (Fujifilm Life Science, USA). Immunoblots were then quantified with ImageJ (NIH) quantification software.

### Statistical analysis

All results are given as mean ± SEM and were considered significant at p ≤ 0.05. The results were analysed with repeated‐measures ANOVA for behavioural tests, one-way ANOVA or unpaired t‐test for tissue analyses. All statistical tests were performed in GraphPad Prism.

### Ethics declarations

All the experiments were carried out in accordance with relevant guidelines and regulations.

## Results

### Diffusion of CDNF in brain tissue

To investigate the possible role of CDNF in HD, we first characterized the diffusion of CDNF in brain tissue after intrastriatal injection (Fig. 1). We observed a wide CDNF diffusion in the striatum, and the substantia nigra pars compacta (SNpc) 2 h after injection. This is in line with the previous data showing that CDNF is retrogradely transported from the striatum to the SNpc^39,51^. Moreover, we observed a widespread diffusion of CDNF throughout the striatum at six hours after infusion. This is in line with recent data showing a half-life of about 5.5 h of CDNF in brain tissues^52^.

### CDNF improves motor coordination and grip strength in QA model in rats

To study the effect of CDNF in a neurotoxin model of HD, we injected PBS as vehicle group (n = 9) or QA in the striatum of rats (225 nM) to mimic the selective loss of GABAergic medium spiny striatal projection neurons. Two weeks later (Fig. 2), rats with QA-lesion received single unilateral injections of CDNF (10 μg) (n = 23) or phosphate-buffered saline (PBS) (n = 23) using the same coordinates as in the diffusion experiments above.

**Figure 3:**
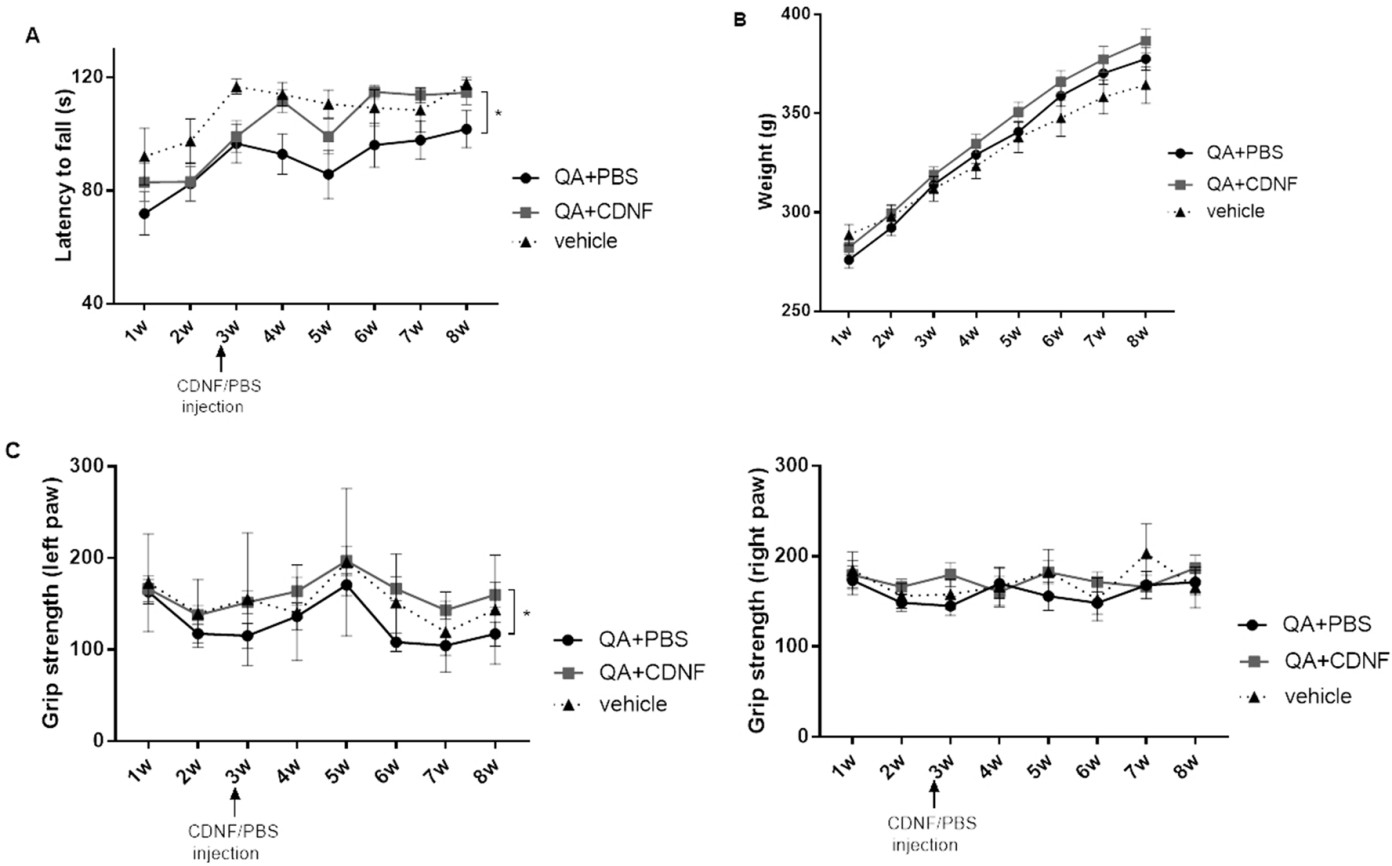
Motor coordination. Rats were treated with vehicle or lesioned by QA and received CDNF- or PBS-treatment into the same location in two weeks post-lesion. (**A**) In Rotarod, CDNF-treated animals showed significant improvement in motor performance in comparison with PBS-treated QA group. Values are expressed as group mean ± SEM. *p < 0.03 vs PBS-treated group, repeated-measures ANOVA. N number per group = 9–23. (**B**) Weight. There was no statistical difference between groups. n = 9–39. (**C**) Grip strength. Performance of CDNF-treated animals was better on the ipsilateral side (left paw) compared with PBS-treated QA group. *p < 0.01 vs PBS-treated group, repeated-measures ANOVA. No difference between groups on the contralateral side (right paw). Values are means ± SEM. N number per group = 9–23.

To assess the motor performance of the animals, we performed the Rotarod test once a week for the rats. Analysis (two-way RM ANOVA) of the results from Rotarod showed a significant treatment effect right after CDNF injection (treatment effect f_2,52_ = 3.631, p = 0.0334; time effect f_7,364_ = 9.248, p < 0.0001; treatment × time interaction f_14,364_ = 0.782, p = 0.6887) (Fig. 3A). There was no significant difference in body weight among groups (Fig. 3B), but the performance in the grip strength test continuously decreased in both groups after 4 weeks post-lesion. Grip strength assay revealed a significant drug treatment effect between PBS- and CDNF-treated rats in the muscular strength of the paw from lesion side (left paw) (two-way RM ANOVA treatment effect f_2,52_ = 4.52, p = 0.015; time effect f_7,364_ = 7.206, p < 0.0001; treatment x time interaction f_14,364_ = 0.7185, p = 0.756). However, the muscle strength of paw showed no effect on the contralateral side between two treatments (Fig. 3C). Results on the balance beam performance test for PBS, control and CDNF-treated animals are shown in Supplemental Fig. 1 with no significant difference between groups.

**Figure 4:**
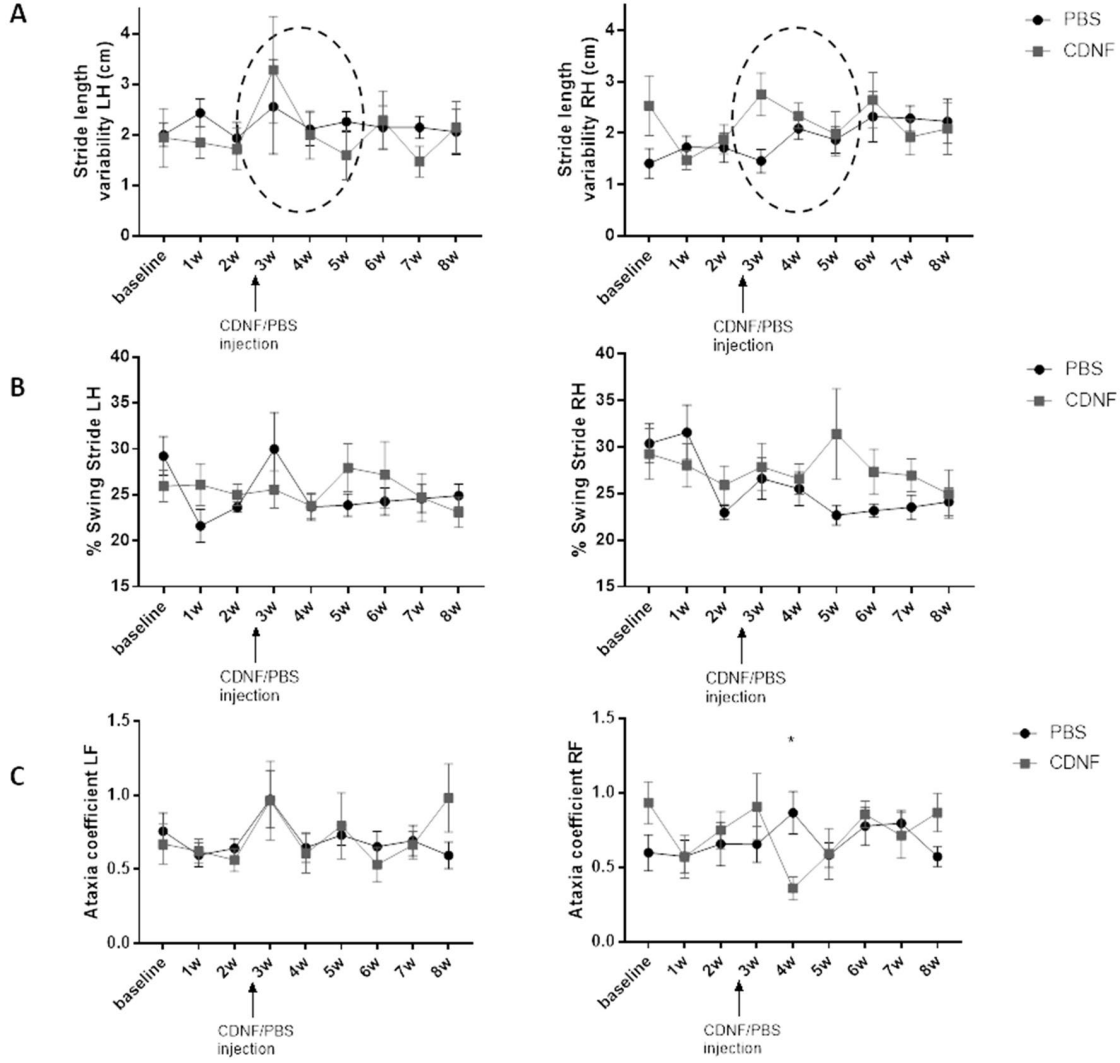
Motor performance in Digigait test. (**A**) Stride length variability. CDNF-treated group shows the decrease in that parameter during 2 week after treatment. (**B**) In the relationship between swing and stride phases there was no difference between groups. (**C**) Ataxia coefficient of the right forelimb (contralateral side) shows a significant decrease in CDNF-treated animals. Values are mean ± SEM. *p = 0.05 vs vehicle, repeated-measures ANOVA, Sidak’s post hoc. N number per group = 7–8. *LH* left hind limb, *RH* right hind limb, *LF* left forelimb, *RF* right forelimb.

Motor activity was investigated further by the use of the treadmill test, DigiGait (Fig. 4). CDNF-treated animals showed a decreased tendency in stride length variability of hind limbs in ipsilateral and contralateral sides at 2-weeks post-treatment. The characteristics in the relation between swing and stride phases fluctuated in the vehicle group, whereas CDNF-treated animals showed more even steps from 1 week to another. Results on the paw area, stride duration and length are shown in Supplemental Fig. 2 with no significant difference between groups. The ataxia coefficient, which is usually increased in HD^53^ was significantly decreased in contralateral forelimb in the CDNF-group (two-way RM ANOVA treatment × time interaction f_8,102_ = 2.002, p = 0.053; treatment effect f_1,102_ = 0.055, p = 0.814; time effect f_8,102_ = 0.939, p = 0.487). The Sidak post hoc test showed a significant difference at 1 week after treatment between PBS- and CDNF-treated rats (p < 0.05). Together these data demonstrate that CDNF significantly improved the motor behaviour performance of QA-lesioned rats.

### CDNF protects striatal neurons in QA-lesion model of HD

**Figure 5:**
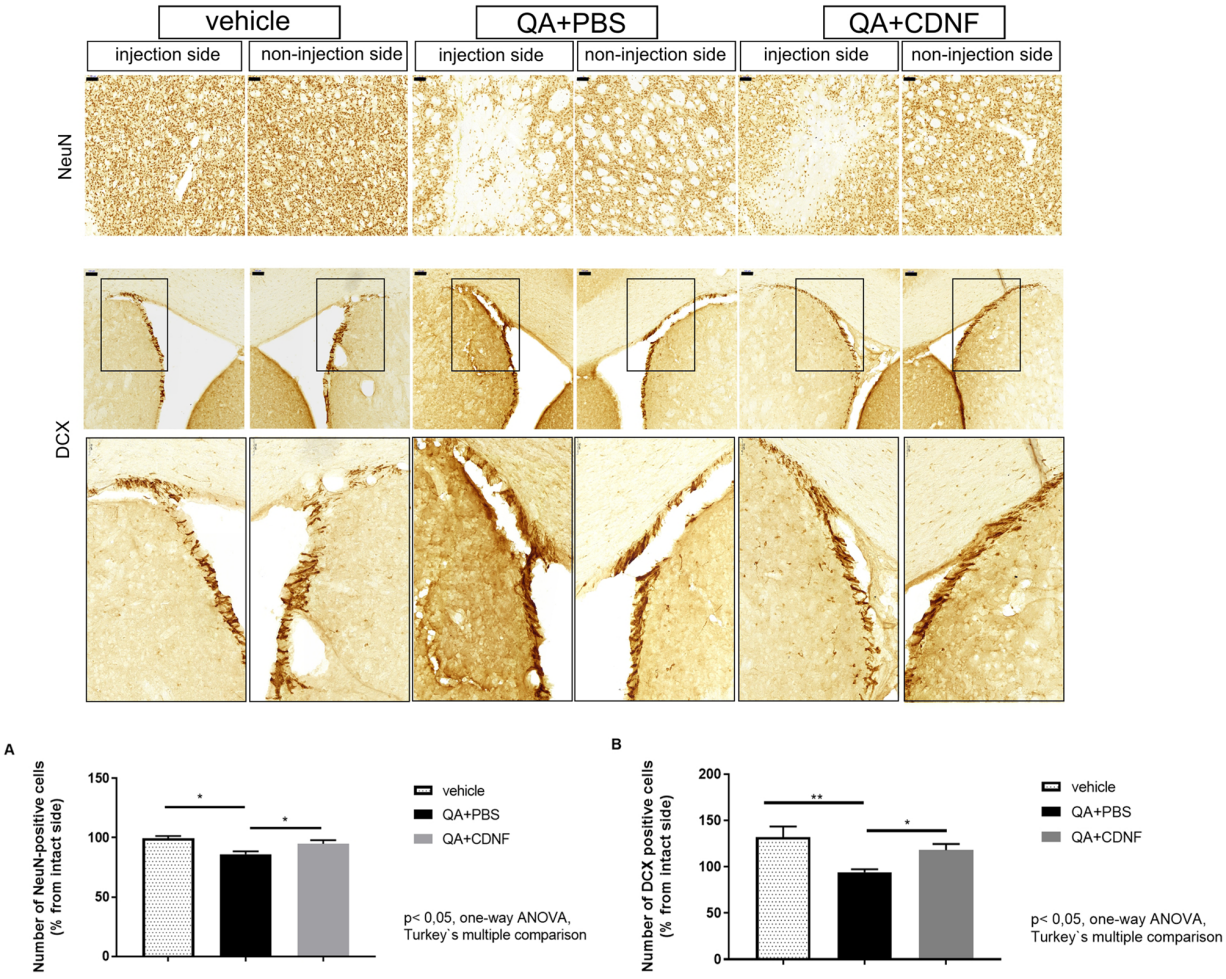
Immunohistochemistry. Coronal sections of striatum show positive NeuN and DCX-immunoreactivity in ventral striatum of QA-lesioned animals after CDNF treatment. Scale bar: 100 μm. Scale bar for bigger magnification: 50 μm. (**A**) Number of NeuN- positive neurons on the left (lesioned) side versus right (non-lesioned) side. (**B**) Number of DCX-positive cells. Immunopositive cells were counted in the 3–5 coronal sections and expressed as mean ± SEM of the total number of cells in the same section (n = 9–23 per group). *p = 0.05, **p = 0.005, one-way ANOVA, Turkey’s post hos test. Untreated controls are set as 100. Images were taken with Pannoramic 250 digital slide scanner (3DHISTECH, Hungary).

Next, we examined whether CDNF can restore features of striatal neurons after intrastriatal QA injections. CDNF did not affect the striatal volume of QA treated rats compared with PBS treatment (Supplemental Fig. S3A). However, the ratio between intact and injected side indicated a positive trend in CDNF-treated group compared to PBS-treated group (Supplemental Fig. S3B). Immunoreactivity was done using antibodies for the NeuN staining neuronal nuclei, or for the microtubule-associated protein, doublecortin (DCX) that is expressed by differentiating neurons and used to detect neurogenesis. QA induced a significant reduction in the number of NeuN-positive cells in the striatum, and CDNF was able to restore these cells (Fig. 5). The analysis was applied by manual calculation in ImagePro software and showed a significant difference between vehicle and QA-lesioned PBS groups (one-way ANOVA, Turkey’s post hoc test p < 0.05), and between QA-lesioned PBS and QA-lesioned CDNF groups (one-way ANOVA, Turkey`s post hoc test p < 0.05) (Fig. 5A).

To study possible changes in differentiating neurons in the striatum by CDNF, the number of DCX-positive cells was quantified at 5 weeks after post-treatment using Matlab software (Fig. 5B). The calculation showed a statistical increase in the number of DCX-positive cells in the striatum of CDNF-treated rats compared with QA-lesioned PBS ones (one-way ANOVA, Turkey’s post hoc test p < 0.05) and between vehicle and QA-lesioned PBS groups (one-way ANOVA, Turkey`s post hoc test p < 0.005) (Fig. 5B).

**Figure 6:**
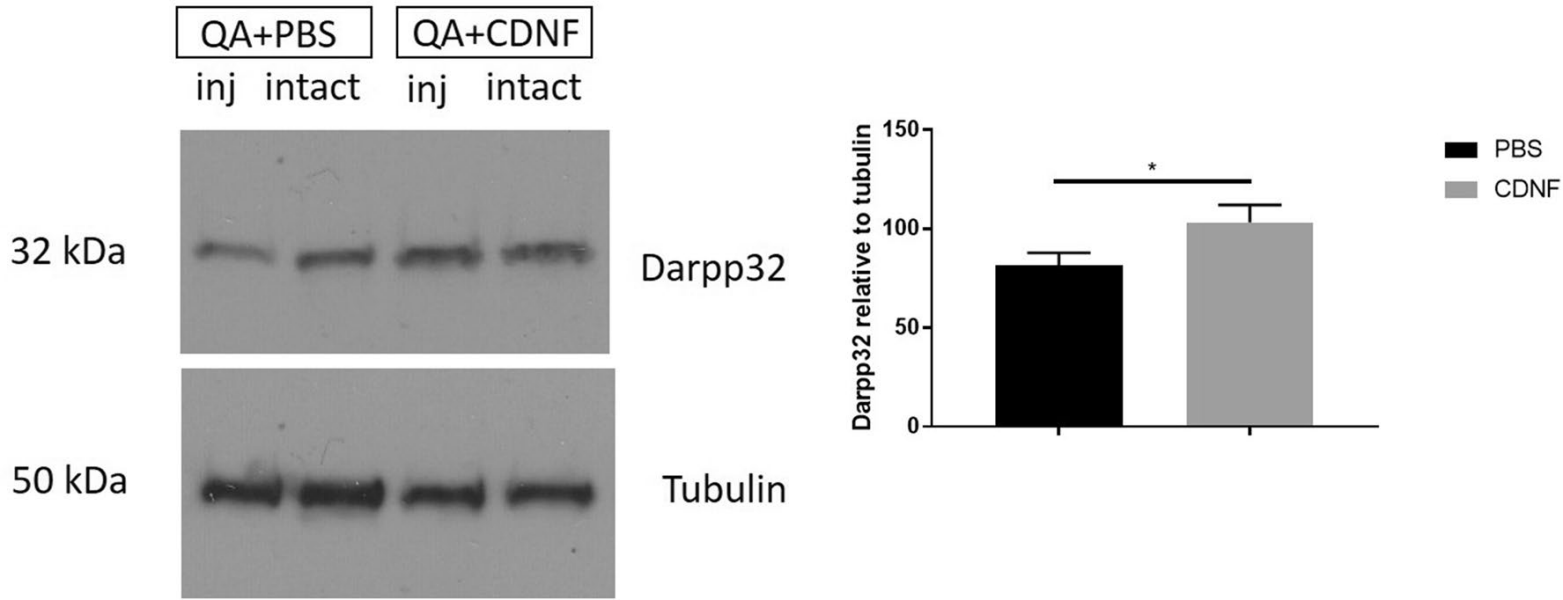
Western blots for Darpp32 after CDNF- and vehicle-treatment in QA-lesioned animals. The density of blots was measured as ratio of injected (inj) and intact side, which were normalized to the loading control. Values are expressed as group mean ± SEM. N number per group = 12–16. *p = 0.05, unpaired Student’s t-test. Untreated controls are set as 100.

Dopamine- and cAMP-regulated phosphoprotein (DARPP-32) is a neuronal protein expressed mainly in the striatum in the MSNs^54^, which are vulnerable in HD. We observed that the expression level of DARPP-32 was reduced in QA-treated rats but significantly increased by CDNF in the striatum compared with controls (PBS, 81.32 ± 6.5; CDNF, 103.2 ± 8.7; *t* = 2.033, *df* = 28, *P* = 0.05, Student’s t-test) (Fig. 6). This demonstrates that CDNF has a beneficial effect on the viability of MSNs that are particularly affected in HD.

### Effects of CDNF in vitro

**Figure 7:**
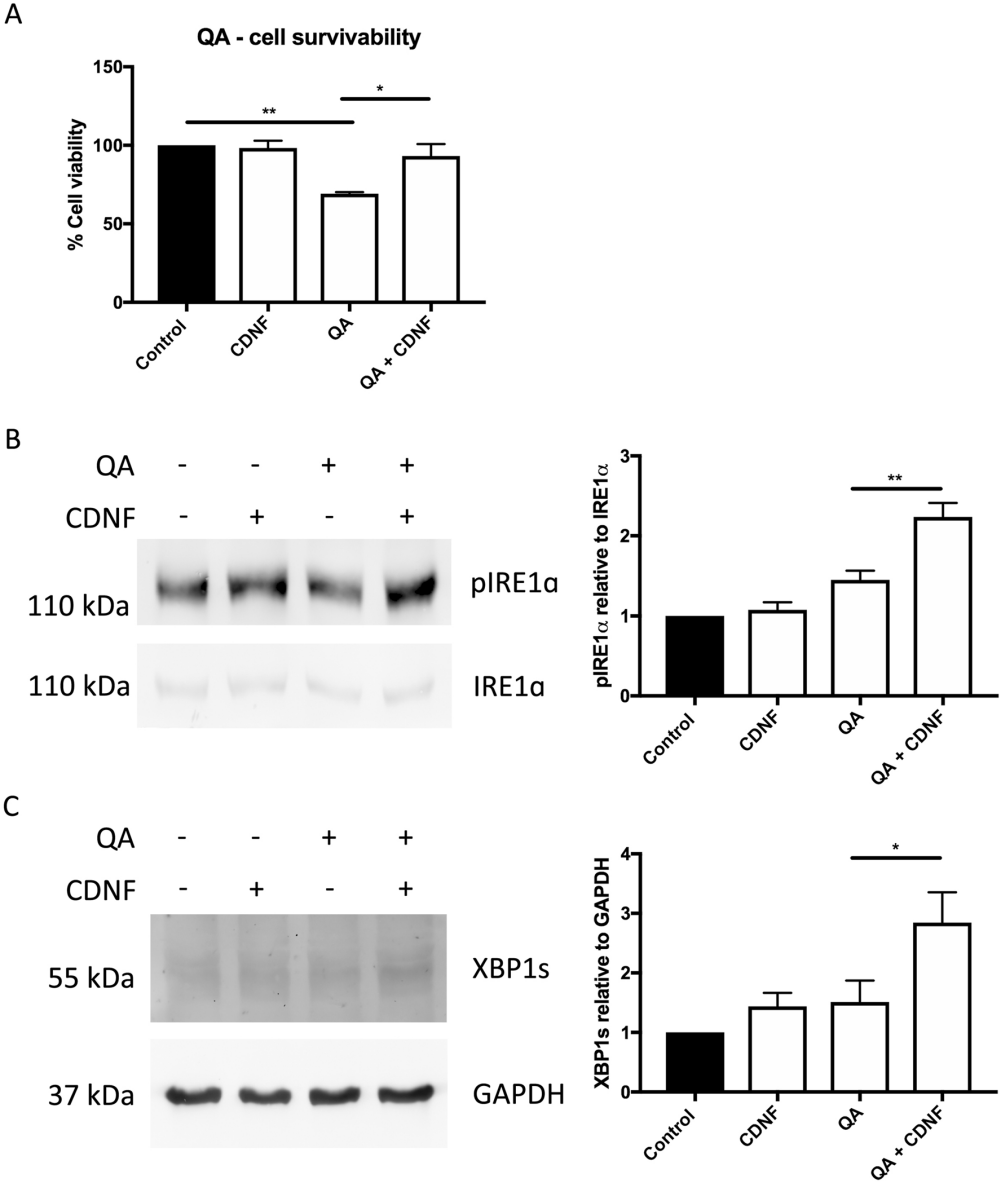
CDNF protects striatal cells against QA toxicity in vitro. Striatal cells were exposed to 3 mM of QA for 24 h. Cell were incubated with and without CDNF protein. Cell viability was evaluated by the MTT assay. *p = 0.02, **p = 0.005, one-way ANOVA, Turkey’s post hoc test. Sample size n = 3. Immunoblot showing the increase in phosphorylated IRE1α (**B**) and spliced XBP1 (**C**) in QA challenged striatal neurons upon treatment with CDNF. Right panels of (**B**,**C**) represents the densitometric ratio of phosphorylated IRE1α to total IRE1α (**B**), and XBP1s to the loading control GAPDH (**C**). Values are expressed as group mean ± SEM. (**B**) **p = 0.0025, n = 3, (**C**) *p = 0.0294, n = 4 one-way ANOVA, Bonferroni’s multiple comparison test.

To study whether CDNF can directly target striatal neurons, we employed cell cultures in which striatal neurons were treated with QA in conjunction with CDNF. Results showed that QA reduced the reactivity/viability of striatal neurons measured in the MTT assay (see “Materials and methods” section) for 24 h by about one-third (one-way ANOVA followed by Turkey’s post hoc test, p < 0.01) (Fig. 7A). The addition of 100 ng/ml CDNF significantly counteracted loss of cell viability induced by QA (one-way ANOVA followed by Turkey’s post hoc test, p < 0.05). The effect of CDNF on cell viability amounted to nearly complete inhibition of the QA-mediated cell loss of striatal neurons. To study the underlying mechanism involved, we employed immunoblotting showing that CDNF enhanced the endoplasmic reticulum stress sensor, IRE1α signalling in the QA challenged striatal neurons. CDNF significantly increased the phosphorylation of IRE1α (one-way ANOVA followed by Bonferroni’s post hoc test, p < 0.005) as well as the level of spliced XBP1 (XBP1s) protein (one-way ANOVA followed by Bonferroni’s post hoc test, p < 0.05) (Fig. 7B,C). Together these results show that CDNF increases the viability of striatal neurons in culture and counteracts the deleterious effects induced by the neurotoxin QA.

## Discussion

The present study demonstrates that CDNF has a survival-promoting effect on striatal neurons in culture when challenged with the neurotoxin, QA. In vivo, a single injection of CDNF rescued striatal neurons from damage and improved motor performance in the QA model of HD. In the following, we will discuss these findings further in view of the beneficial effects observed with CDNF in the QA model.

The QA neurotoxin model mimics alterations observed in patients afflicted by HD concerning behavioural and neuropathological features^55^. Thus, our results are promising in revealing positive effects of CDNF in the QA model by significantly improving the motor performance as shown in the Rotarod test and improve motor deficits as shown in the grip strength test. Moreover, CDNF decreased the ataxia coefficient in Digigait test, and gait, which is part of the pathology of HD. CDNF was able to protect QA- induced lesions in the striatum. We suggest that these positive effects could be associated with the ability of CDNF to diffuse widely in the striatum, as observed in this study.

The main characteristic of HD pathology is the loss of MSNs in the striatum. QA as a neurotoxin induces damage of MSNs when used in experimental animals^56^. QA is present in human and rat brains in nanomolar concentrations^57^ but is increased by 300–400% in HD brains^58^. It is known that QA has multiple effects in brain tissue, including effects on microglia and neurons themselves. In the present study, we observed overt degeneration of neurons in the striatum after local injection of QA. Immunohistochemistry showed a reduced number of NeuN-immunopositive striatal neurons after QA administration and CDNF protected NeuN positive cells. This suggests that CDNF has a beneficial effect on neuronal viability after QA treatments. In addition, the protein level of DARPP-32 was increased by CDNF in the QA-treated striatum, indicating that CDNF may rescue DARPP-32 positive MSNs. In line with this, experiments with striatal neuronal cultures demonstrated that CDNF protects against QA-mediated cell demise in vitro. The addition of CDNF counteracted the loss of striatal neuron viability induced by QA indicating a direct effect of the factor on these neurons in culture. Functionally, we observed that the IRE1α/XBP1 signalling in the ER was enhanced by the addition of CDNF to QA-treated striatal cells. Previous studies have demonstrated that the expression of spliced form of the transcription factor XBP1 (XBP1s) is neuroprotective in models of HD^42^. In line with this, we observed that CDNF increased the levels of spliced XBP1 (XBP1s) in cultured striatal cells challenged with QA. The precise genes and proteins downstream of XBP1s involved in the action of CDNF in striatal cells warrants further investigation in the future. Our results are supported by findings that CDNF reduces the ER stress markers level in vitro and in vivo in PD models^37^. We postulate that the beneficial effects of CDNF in the QA model may be associated with an increase in IRE1α/XBP1 ER signalling by mechanisms to be explored.

A further interesting aspect of this study was that the number of DXC-immunoreactive cells was increased in rat striatum by a single injection of CDNF. This may be interpreted that CDNF could possibly increase the number of DXC-positive differentiating neurons in the QA-lesioned striatum. Previous studies have shown that striatal injury can induce neurogenesis in areas close to the subventricular zone^59,60^, and influence the migration of neuronal precursors into the damaged striatum^61^. Most noteworthy, neurogenesis has also been reported in the human brain, afflicted by HD^62^.

In the present study, we observed an increase of DXC-positive cells also in the sham group, which is supported by the previous finding of QA-induced migration and formation of new neurons in the lesioned striatum. At the same time, we found that CDNF increases the DCX-immunoreactivity, which is the marker for active neurogenesis. These results are in line with the previous study^63^ demonstrating that mesencephalic astrocyte-derived neurotrophic factor MANF, the second member of CDNF/MANF neurotrophic factor family, can promote migration of DCX-positive cells in the infarct area in a rat model of stroke. Furthermore, MANF was reported to enhance the production of newly generated neurons^64–66^. It remains to be studied whether CDNF may increase either neurogenesis or the differentiation of neuroblasts in HD, as well as in other types of brain disorders.

Taken together, this study demonstrates that the neuroprotective effects of CDNF can be associated with enhanced neurogenesis, as evident using a single injection of this factor in the animal model of HD. These results on neurogenesis in the QA model warrant further studies using different times of treatment and doses of CDNF in genetic models of HD.

## Conclusions

We report here beneficial effects of a single intrastriatal injection of CDNF on neuronal viability and behaviour parameters in a QA neurotoxin model of HD. The precise mechanisms underlying the action of CDNF in the striatum requires further investigations, but the data indicates a potential protective function and neurogenesis stimulation of this molecule in the QA-lesioned striatum. Moreover, we hypothesize that the neuroprotective effect of CDNF can be explained by the influence over IRE1α/XBP1 signalling. Neuroprotective effects of CDNF have been described in other models of neurodegenerative diseases, including PD^37^. The present results indicate a potential beneficial role of CDNF in HD and can be considered as a promising factor for designing better treatment against the disease in the future. We are currently investigating the role of CDNF in genetic models of HD as well as the signalling pathways affected by this factor in the striatal neurons.

## Supplementary information

Supplementary Information.
