# Supplementary material for: Cerebral dopamine neurotrophic factor (CDNF) protects against quinolinic acid-induced toxicity in in vitro and in vivo models of Huntington’s disease
Source: Sci Rep. 2020 Nov 5;10:19045. doi: 10.1038/s41598-020-75439-1 (PMC7645584; doi:10.1038/s41598-020-75439-1)

Cerebral dopamine neurotrophic factor (CDNF) protects against quinolinic acid-induced toxicity *in vitro* and *in vivo* in a model of Huntington's disease

Stepanova P[1], Srinivasan V[2,3], Lindholm D[2,3], and Voutilainen MH[1].

[1]Institute of Biotechnology, HiLIFE, University of Helsinki, Helsinki, Finland. BO Box 56, FIN-00014, University of Helsinki, Finland

[2]Medicum, Department of Biochemistry and Developmental Biology, Faculty of Medicine, POBox 63, FIN-00014 University of Helsinki, Finland

[3]Minerva Foundation Institute for Medical Research, Biomedicum Helsinki 2U, Tukholmankatu 8

Supplementary information

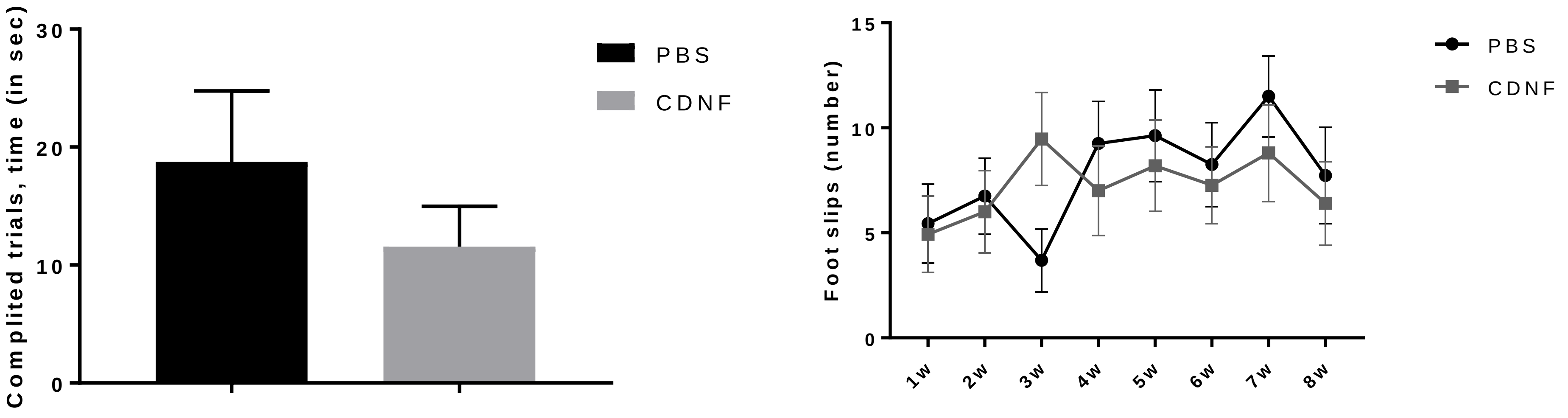

Supplementary Fig 1. Balance beam test. Rats were lesioned by QA and received CDNF- or PBS-treatment into the same location in two weeks post-lesion. Values are expressed as group mean ± SEM. No difference between groups in travel time and foot slips number. N number = 15-16.

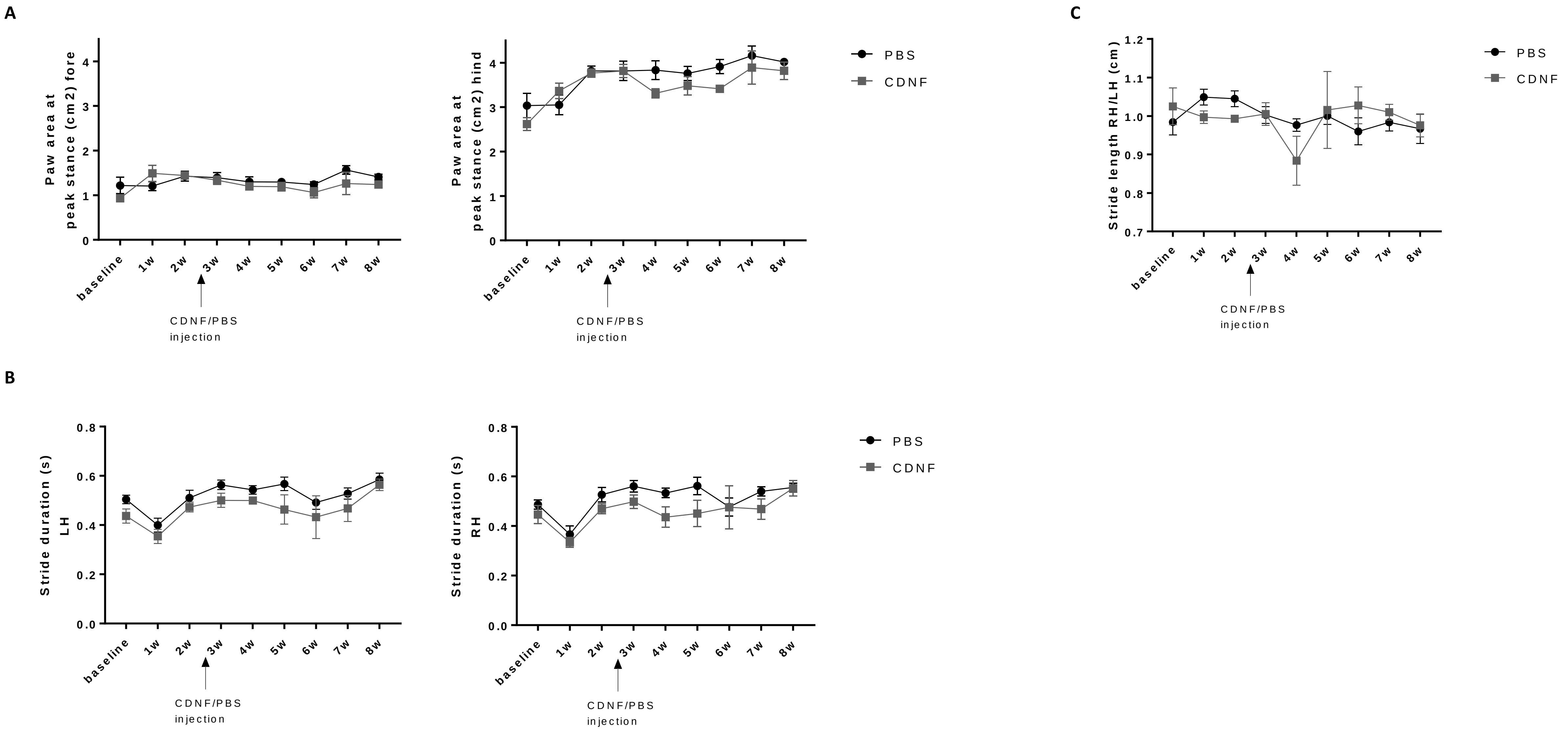

Supplementary Fig 2. (A) Paw area of forelimbs (fore) and hind limbs (hind). (B) Stride duration of lesion side hind limb (LH) and intact side hind limb (RH) In the relationship between swing and stride phases there was no difference between groups. (C) Stride length. Ratio between intact side (RH) and lesion side (LH). Values are mean ± SEM. N number per group = 7-8. Left hind limb (LH), right hind limb (RH).

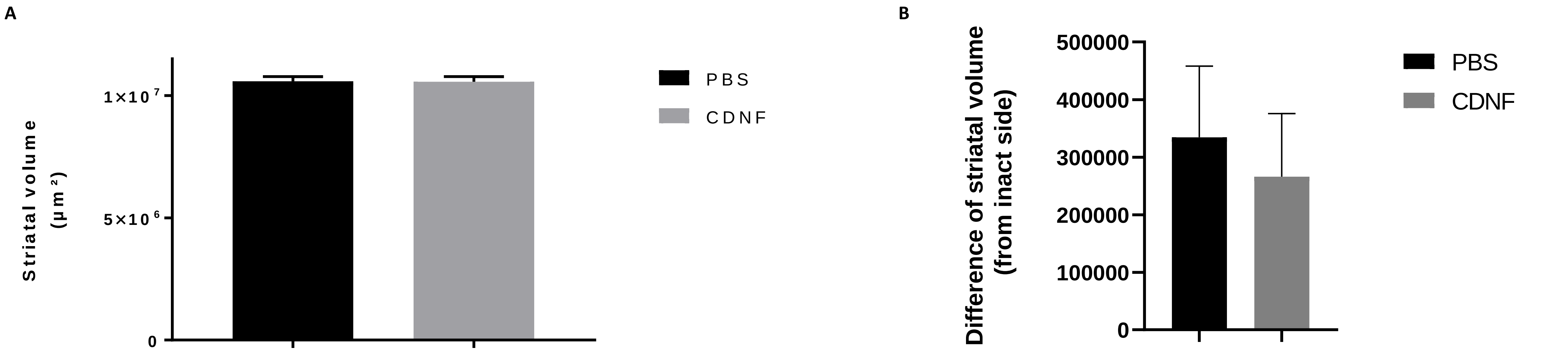

Supplementary Fig 3. Striatal volume. (A) Quantification of the area of QA-lesioned striatum in CDNF- and PBS-treated groups . (B) The difference of striatal volume between intact and injected sides. There was no statistical difference between groups. Values are expressed as group mean ± SEM. N number n=19-23 per group.

Supplementary Fig 4. Western blots. Full blots for representative figure 6. Scale repeats the scale of Dual Color marker.

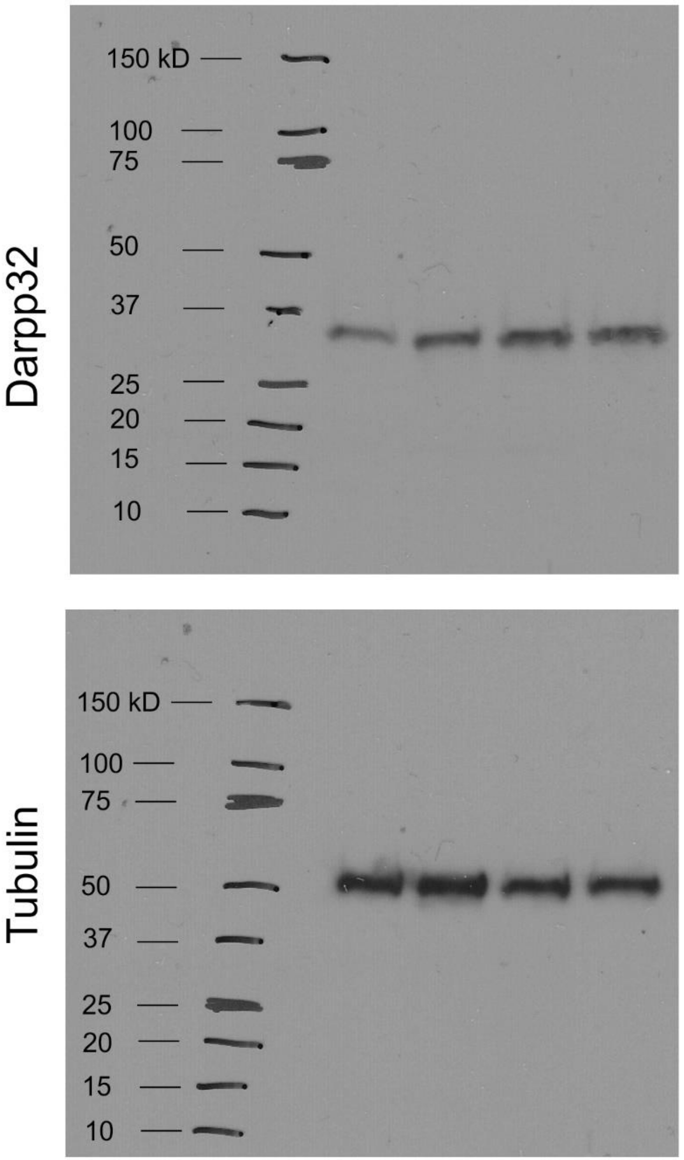

Supplementary figure 5. - Full blots for representative figure 7B and 7C

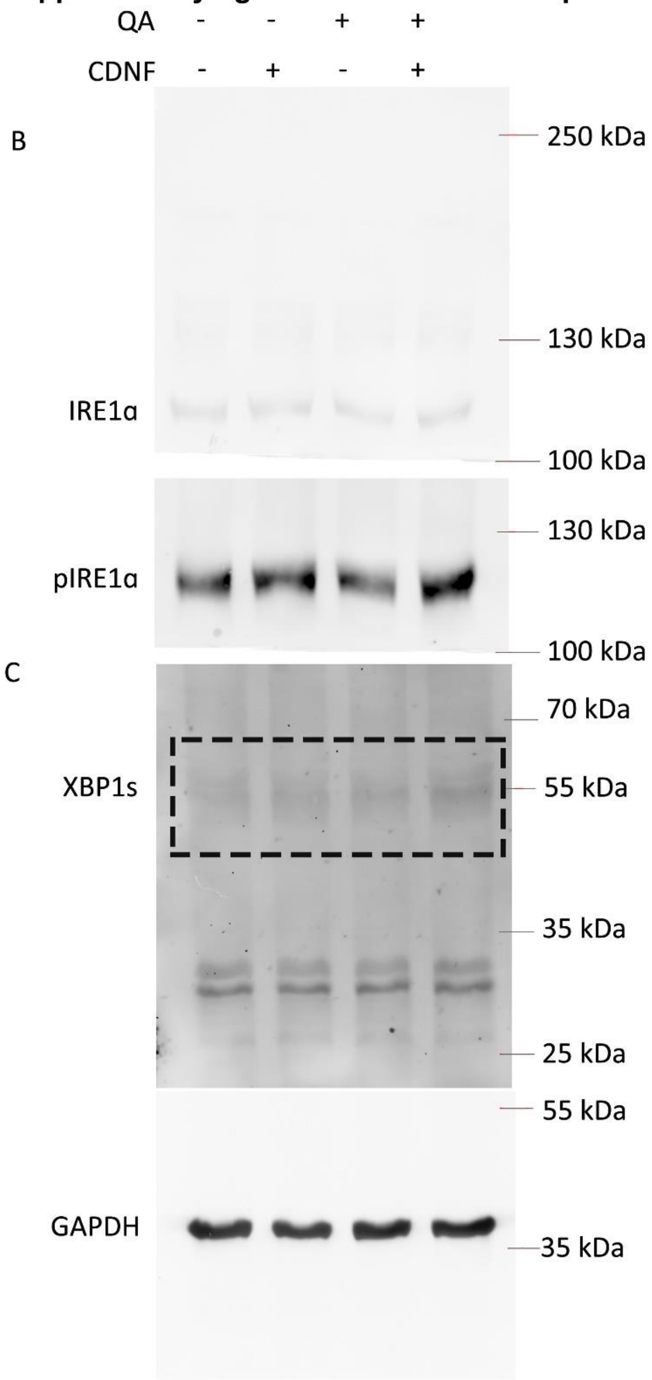

Supplement: Supplementary file 1 — Supplementary Information. [file 41598_2020_75439_MOESM1_ESM.pdf]
